# APC-Cdh1 Inhibits the Proliferation and Activation of Oligodendrocyte Precursor Cells after Mechanical Stretch Injury

**DOI:** 10.1155/2019/9524561

**Published:** 2019-04-17

**Authors:** Xiang Ma, Yongqing Guo, Yuehong Qi, Hongpeng Zhang, Yuhua Hao, Huaping Zhang, Xiaona Li

**Affiliations:** ^1^Department of Anesthesiology, Shanxi Medical University, Taiyuan, China; ^2^Department of Anesthesiology, The People's Hospital of Shanxi Medical University, Taiyuan, China; ^3^Translational Medicine Research Center, Shanxi Medical University, Taiyuan, China; ^4^Institute of Applied Mechanics and Biomedical Engineering, Taiyuan University of Technology, Taiyuan, China

## Abstract

The incidence of spinal cord injury (SCI) continues to increase; however, the involved mechanisms remain unclear. Anaphase promoting complex (APC) and its regulatory subunit Cdh1 play important roles in the growth, development, and repair of the central nervous system (CNS). Cdh1 is involved in the pathophysiological processes of neuronal apoptosis and astrocyte-reactive proliferation after ischemic brain injury, whereas the role played by APC-Cdh1 in the proliferation and activation of oligodendrocyte precursor cells (OPCs) after SCI remains unresolved. Using primary cultures of spinal oligodendrocyte precursor cells, we successfully established an* in vitro* mechanical stretch injury model to simulate SCI. Cell viability and proliferation were determined by MTT assay and flow cytometric analysis of the cell cycle. Real-time fluorescent quantitative PCR and Western blot analysis determined the mRNA and protein expression levels of Cdh1 and its downstream substrates Skp2 and Id2. Mechanical stretch injury decreased the proliferative activity of OPCs and enhanced cellular Cdh1 expression. Dampened expression of Cdh1 in primary OPCs significantly promoted proliferation and activation of OPCs after SCI. In addition, the expression of the downstream substrates of Cdh1, Skp2, and Id2 was decreased following mechanical injury, whereas adenovirus-mediated Cdh1 RNA interference increased postinjury expression of Skp2 and Id2. These findings suggest that APC-Cdh1 might be involved in regulating the proliferation and activation of OPCs after mechanical SCI. Moreover, degraded ubiquitination of the downstream substrates Skp2 and Id2 might play an important role, at least in part, in the beneficial effects of OPCs activity following SCI.

## 1. Introduction

Spinal cord injury (SCI) is a disabling and traumatic disease of the central nervous system (CNS), which often causes permanent and irreversible loss of function in afflicted patients. It was suggested that oligodendrocyte precursor cells (OPCs), which are located in the white matter of the CNS, could rapidly respond to SCI and then proliferate and differentiate into mature oligodendrocytes (OLs) with a myelin-forming capability [1]. The proliferation, activation, and differentiation of OPCs after SCI is regulated by many factors that play a critical role in the processes of subsequent axonal remyelination [2]. However, the specific mechanisms that are involved in regulating the proliferation and activation of OPCs after SCI have not been fully elucidated.

The large multimeric E3 ubiquitin ligase Anaphase Promoting Complex (APC) is recognized as one of the main E3 ubiquitin protease systems in living cells that serves a pivotal role in controlling sister chromatid segregation and cellular exit from mitosis [3]. APC helps drive the degradation of protein regulator of the cell cycle, a complex system of molecular events that collectively coordinate chromosome replication and segregation with cell division and growth [4]. Moreover, Cdh1 is an APC coactivator that directly binds to substrates of the APC [4]. Acting as the key regulatory APC subunit, it is also recognized that Cdh1 is important in targeted degradation and does so by specifically binding to the downstream substrates Skp2, Id2, SnoN, and CyclinB1, among others [5].

In recent years, it was found that Cdh1 was highly expressed in neurons and that APC-Cdh1 was involved in many fundamental life activities of the CNS, including axonal elongation, neuronal survival, differentiation, glucose metabolism, and even glial cell proliferation. APC-Cdh1 is also an important regulator of the growth and development of the CNS [6]. Previous work has shown that APC-Cdh1 also plays an important role in CNS injury, and its abnormal activity is an important cause of neuronal apoptosis and astrocyte-reactive proliferation after ischemic brain injury [7, 8]. In addition, APC was identified as the major ubiquitin protease system in cells, and together with its regulatory subunit Cdh1 constituted the APC-Cdh1 pathway, which serves an important intracellular mechanism to negatively regulate the cell cycle [9]. Thus, we speculated that APC-Cdh1 might also influence the proliferation and activation of OPCs following SCI. By regulating the activity of APC-Cdh1, it might be possible to promote the activation and regeneration of OPCs following CNS injury.

## 2. Materials and Methods

### 2.1. Isolation, Purification, and Culture of Spinal Cord-Derived OPCs

Within 48 hours, newborn spinal cord tissues of Sprague–Dawley rats were selected and the primary rat spinal OPCs were cultured* in vitro* [10]. The isolated spinal cord tissues were prepared under a dissecting microscope and sliced into small pieces at approximately 1 mm^3^. The pieces were digested with trypsin for 15 min and then the digestion was neutralized. The liberated cells were resuspended in DMEM culture medium (Gibco, USA) containing 20 percent fetal bovine serum and then seeded into a 75 cm^2^ culture flask that had been precoated with poly L-lysine (0.1 mg/mL; Sigma). The cells were cultured in a 37°C, five percent CO_2_ incubator with the culture medium being changed every three days. When cultured for 10 days, a large number of OPCs were overlaid onto the superior layer of the astrocytes. The culture flask was fixed on a horizontal shaker at a constant temperature of 37°C and then preshaken for one hour to remove the microglia, following which, the culture continued to be shaken at 200 rpm overnight. The cell culture supernatant was then reseeded into an uncoated culture dish for 40 min to remove excess astrocytes and microglia, so that the purified OPCs could be obtained. Single cell suspensions of OPCs were prepared using oligodendrocyte precursor cell culture medium or OPCM (supplemented with PDGF-AA and bFGF) and seeded at a density of 1 × 10^4^/cm^2^ onto a BioFlex VI six-well plate (Flexcell, USA) that was coated with poly L-lysine and collagen I. The purified OPCs were further cultured for three days, after which, subsequent experiments were performed. Immunofluorescence staining showed that more than 95 percent of cells expressed the OPC-specific antigen A2B5. All animal-related procedures in this study were conducted in accord with published regulations (Guide for the Care and Use of Laboratory Animals, 8th edition, NRC, USA) and approved by the local Ethics Committee of Shanxi Medical University.

### 2.2. Construction of Mechanical Stretching Injury Model of OPCs

The mechanical stretch injury model of OPCs was designed according to the methods published in a prior study [11]. Briefly, BioFlex six-well plates were seeded with OPCs that then connected to the FX-4000T™ flexible substrate stretching system (Flexcell, USA) to perform uniform periodic mechanical stretching of the OPCs. The loading parameters were as follows: stretching amplitude of 10 percent; frequency of 0.1 Hz; and a waveform that was a sine wave. The cells were loaded for different durations according to the experimental groupings. After loading into the stretch culture system and completing the culture, the cells were harvested after 48 hours of static culture. Control cells were also seeded and cultured in the BioFlex six-well plate system and subjected to the same environmental conditions as described for the injury group (37°C, 5% CO_2_), but in the absence of being connected to the stretching system. The degree of cell injury was measured by an Annexin V Apoptosis Detection Kit assay (Sangon, China) as determined by flow cytometry (BD, USA). The apoptotic rate in the injured group was significantly increased and the cells did not demonstrate any obvious shedding as compared with those in the control group. This observation suggested that the mechanical stretching injury model of oligodendrocyte precursor cells was successfully constructed.

### 2.3. Adenovirus Transduction of Oligodendrocyte Precursor Cells

The Cdh1-shRNA adenoviral vector construct was obtained from Hanheng Biotechnology Co., Ltd. The Cdh1-shRNA adenoviral construct and the empty vector adenoviral construct were separately added to the cells at a multiplicity of infection (MOI) = 60. The system was subsequently changed to normal culture medium for continuous culture after a transduction time of eight hours at 37°C. After 72 hours of transduction, total RNA and total protein were extracted with the purpose of identifying the effects of Cdh1 RNA interference. Cells that had been transduced with the Cdh1-shRNA construct for 72 hours were subjected to stretch injury for 12 hours, and as described above.

### 2.4. MTT Assay

The MTT colorimetric assay was used for the determination of cell viability. Briefly, a density of 2′10^5^ OPCs was seeded into a 96-well plate and was cultured for 48 hours. After adding 150 ul of MTT solution to each well, incubation of the plate was continued for 4 hours at 37°C in a five percent CO_2_ incubator. After termination of the culture, the supernatant was discarded. Next, 150 ul of DMSO was added to each well to solubilize the purple/blue formazan crystals, and the resultant colored solution was shaken at 37°C for 15 min to completely dissolve the formazan crystals. The formazan solution was then transferred to a 96-well plate, and the OD absorbance values of each group were measured at a wavelength of 570 nm using a microplate reader (Bio-Rad, USA) to reflect viable cell proliferation.

### 2.5. Cell Cycle

After cells were seeded and cultured for 48 hours, the cells were harvested and fixed in 70 percent ice-cold ethanol at 4°C overnight. Next, stages of the cell cycle were estimated according to the manufacturer's instructions (KeyGen, China). The ModFit-LT software was used to analyze the frequency of cells that had compartmentalized to each phase of the cell cycle. Cell proliferation was estimated by measuring the proportion of cells that occupied the S phase by flow cytometry.

### 2.6. Real-Time PCR

Following treatment, total RNA was extracted with Trizol reagent (Invitrogen, USA), and changes in mRNA expression of Cdh1 and its downstream substrates were quantified by a two-step qRT-PCR. Total RNA was reverse transcribed using a Reverse Transcription Kit (Fermentas, USA), following which PCR was carried out on a StepOnePlus™ real-time fluorescence quantitative PCR system using SybrGreen I as the fluorescent dye. All the primers in this experiment (see Table 1) were designed and synthesized by TAKARA. *β*-actin was used as an internal reference control and amplified with the genes of interest and detected in the same reaction system. PCR conditions were as follows: predenaturation at 94°C for 10 min, activation of Taq enzyme; denaturation at 94°C for 15s, annealing at 60°C, and extension for 60s, at a total of 40 cycles. The relative mRNA expression levels of Cdh1 and its downstream substrates, Skp2 and Id2, were analyzed by the 2^−ΔΔCT^ method.

### 2.7. Western Blot

Following treatment, the cells were fully lysed on ice with PMSF-containing RIPA lysate buffer (Beyotime, China), and total cellular protein was extracted. After sample quantification of total protein, all samples were equilibrated to the same concentration with RIPA lysate buffer. An equal volume of boiled denatured protein was taken and separated by 10 percent SDS-PAGE, after which, the protein was transferred to a methanol pretreated PVDF membrane. After blocking with a five percent BSA nonspecific protein solution, a Cdh1 polyclonal antibody (1:2000, Abcam, UK), a Skp2 monoclonal antibody (1:1500, Abcam, UK), an Id2 monoclonal antibody (1:1700, Abcam, UK), and the *β*-actin monoclonal antibody (1:1000, Santa Cruz, USA) were added to the blotted membranes and incubated overnight at 4°C. Next day, the membrane was rinsed and incubated with horseradish peroxidase-labeled secondary antibody (1:6000, Abcam, UK) for 2 h at room temperature. Development of the antibody probed membranes by enhanced chemiluminescence (ECL) was performed after three rinses in TBST buffer. Photographic imaging of the developed membranes was performed using the Bio-Rad ChemiDoc MP versatile gel imaging analysis system. The relative expression levels of Cdh1, Skp2, and Id2 proteins were corrected by using *β*-actin as an internal reference control.

### 2.8. Statistical Analysis

Statistical analysis was performed using SPSS version 17.0 software. The measured data was expressed as mean ± standard deviation (mean ± SD). An independent sample Student's t-test was used to compare between two groups of data. One-way ANOVA compared observations between multiple groups, and Tukey's posttest was used to correct for multiple comparisons between and within groups. The test level was set at an alpha value of P<0.05, which was considered a statistically significant difference.

## 3. Results

### 3.1. Decrease in OPCs Proliferation and Coordinate Increase in Expression of Intracellular Cdh1 after Mechanical Stretching

**Figure 1:**
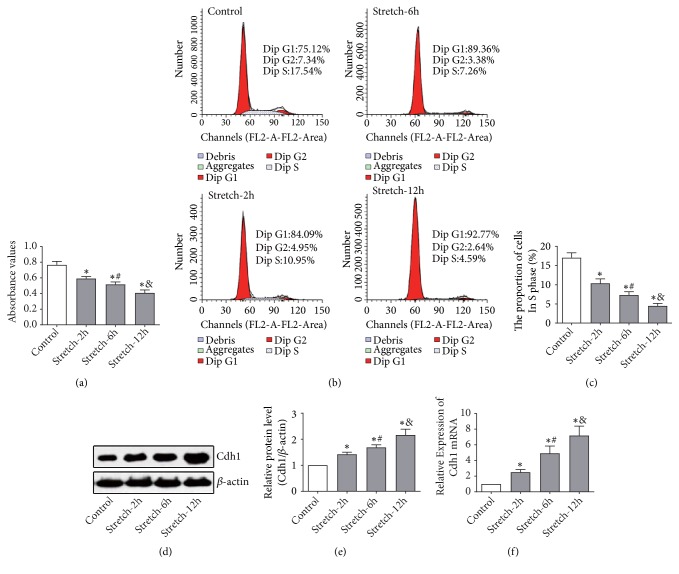
Mechanical stretching-induced decreases in OPCs proliferation and enhanced expression of cellular Cdh1. Purified OPCs were seeded into BioFlex six-well plates and connected to a stretching system to conduct mechanical injury of the cells, which were statically cultured for 48 h after being loaded according to the treatment grouping. (a) MTT detection of cell viability after mechanical injury. The OD value of the injured group gradually decreased with the extension of stretching time and was lower as compared with the control group. (b, c) Flow cytometric analysis of the effects of mechanical injury on OPCs cell cycle distribution. The proportion of injury group cells in S phase was significantly decreased as compared the control group, and it had a tendency of decrease with time. (d, e) Effects of mechanical stretch injury on protein expression of Cdh1 in OPCs. Western blot analysis showed increased protein expression of Cdh1 in the injury group as compared the control group. Additionally, expression of Cdh1 increased with progression of mechanical stretching time. Relative expression levels were determined by densitometry and normalized to *β*-actin expression as an internal reference. (f) Effects of mechanical stretch injury on Cdh1 mRNA expression in OPCs. Real-time fluorescent quantitative PCR analysis suggested that, compared with the control group, the Cdh1 mRNA expression in the injury group was significantly increased, and it had a tendency of increase with the extension of mechanical stretch time. N = 3; *∗*P < 0.05 versus Control; #P < 0.05 versus Stretch at 2 hours; and P < 0.05 versus Stretch at 6 hours.

By MTT cell viability assay and cell cycle analysis, we analyzed the effects of mechanical injury on the proliferation of OPCs. Cell viability began to decrease after two hours of mechanical stretching, and the cell viability of the injury group decreased as a function of extending the stretching time, and especially in the Stretch-12 hours group (P<0. 05; Figure 1(a)). Cell cycle analysis showed similar results. The proportion of injury group cells in the S phase of the cell cycle gradually and significantly decreased as a function of extended stretch time, which was lower than found in the control group (P<0.05; Figures 1(b) and 1(c)). Observations suggested that mechanical stretching inhibited OPCs proliferation in a time-dependent manner, which was most obvious following 12 hours of stretch. Western blot analysis and real-time PCR analysis showed that mechanical stretching increased Cdh1 expression in a time-dependent manner (Figures 1(d)–1(f)). These findings suggest that mechanical stretching of OPCs decreases cell proliferation, which is accompanied by enhanced expression of intracellular Cdh1 and stretch at 12 hours was used for subsequent analysis.

### 3.2. Effectiveness of an Adenoviral Vector to Silence Cdh1 RNA Expression

**Figure 2:**
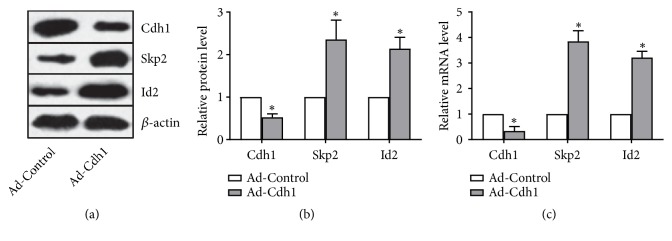
Identification of the effectiveness of adenoviral vector-mediated interference of Cdh1 RNA expression. The adenovirus was diluted, and the Cdh1-shRNA adenovirus construct and the empty adenovirus vector were separately added to the cells at an MOI = 60, following which, cells were harvested 72 hours after transduction. (a, b) Effects of Cdh1-shRNA adenovirus transduction on Cdh1 expression and its downstream protein substrates Skp2 and Id2 in OPCs. Western blot analysis demonstrated results showed that the protein expression of Cdh1 in OPCs was significantly decreased as compared the empty vector in the control group; additionally, the protein expression of Skp2 and Id2 was significantly increased after Cdh1-shRNA adenovirus transduction. (c) Cdh1 expression and its downstream substrates Skp2 and Id2 mRNA after Cdh1-shRNA adenovirus transduction. Real-time PCR analysis showed that Cdh1 mRNA expression in OPCs was significantly decreased as compared transduction with the empty vector in the control group; further, Skp2 and Id2 mRNA expression was significantly increased after Cdh1-shRNA adenovirus transduction. N = 3; *∗*P < 0.05 versus Ad-Control.

To confirm the effectiveness of a Cdh1-shRNA adenoviral vector in silencing Cdh1 expression, OPCs were collected 72 hours after adenoviral transduction, following which the expression of Cdh1 was determined by RT-PCR and Western blot analysis (Figures 2(a)–2(c)). Compared with the vehicle group, the Cdh1-shRNA adenoviral construct significantly decreased Cdh1 (all P<0.05). In addition, changes in expression of the Cdh1 downstream substrates Skp2 and Id2 were also detected after interference. Observations demonstrated that the mRNA and protein expression levels of Skp2 and Id2 exceeded those seen in the empty virus group (P<0.05, respectively). This observation contrasted with the observed decrease in Cdh1 expression (Figures 2(a)–2(c)).

### 3.3. Cdh1 Knockdown Promoted OPCs Proliferation and Activation after Mechanical Stretching

**Figure 3:**
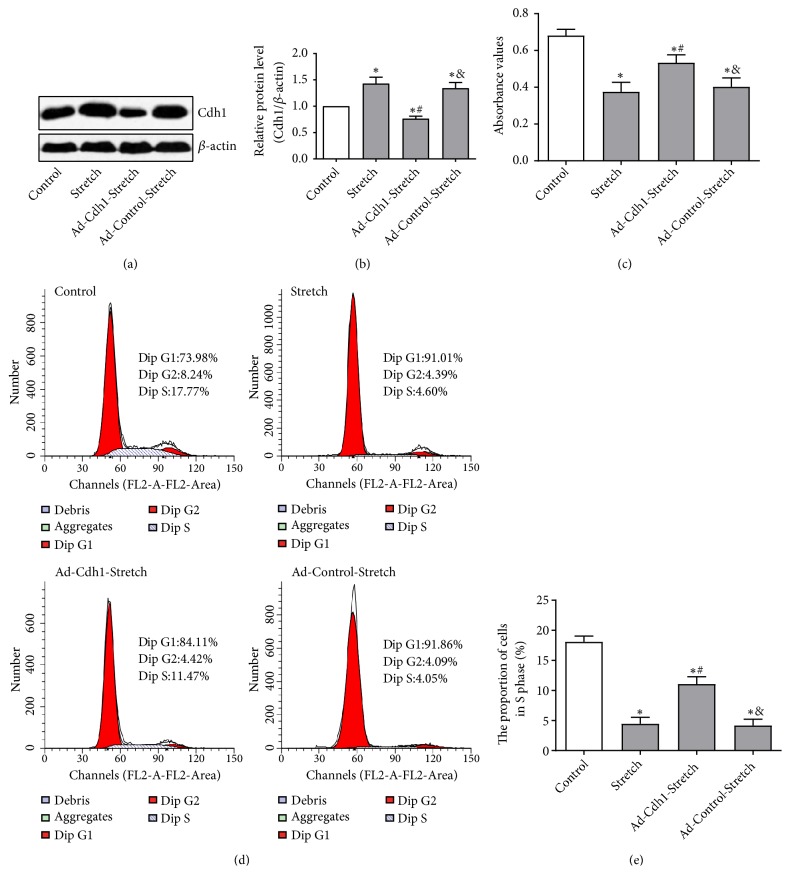
Adenoviral-mediated Cdh1 RNA interference promoted OPCs proliferation and activation following mechanical stretching injury. After OPCs were respectively infected for 72 hours with Cdh1-shRNA adenovirus and the empty adenoviral vector, the mechanical stretching injury was applied for 12 hours. After completing the loading, static culture was continued for another 48 hours. (a, b) Cdh1 protein expression levels in the Control, Stretch, Ad-Cdh1-Stretch, and Ad-Control-Stretch groups are shown. Western blot analysis showed that, compared with the Ad-Control-Stretch group, adenoviral-mediated Cdh1 RNA interference significantly downregulated Cdh1 protein expression in OPCs after mechanical injury. (c) MTT assay of cell viability. Compared with Ad-Control-Stretch group, the OD value of the Ad-Control-Stretch group was significantly increased but was not at the level seen in the normal control group. (d, e) Flow cytometry of cell cycle distribution. The proportion of S phase cells in the Ad-Cdh1-Stretch group was significantly higher than that seen in the Ad-Control-Stretch group. N = 3; *∗*P < 0.05 versus Control; #P < 0.05 versus Stretch; and P < 0.05 versus Ad-Cdh1-Stretch.

Next, the important role played by APC-Cdh1 in the proliferation and activation of OPCs after mechanical stretch injury was explored by knocking-out Cdh1 expression by RNA interference and they were divided to Control, Stretch, Ad-Control-Stretch, and Ad-Cdh1-Stretch groups. Western bolt analysis confirmed that Cdh1 protein expression in the Ad-Cdh1-Stretch group was significantly lower than the Ad-Control-Stretch group (P<0. 05). This observation indicated that Cdh1 knockdown significantly inhibited the ability of mechanical stretch to alter Cdh1 protein expression (Figures 3(a) and 3(b)). Next, the effect of Cdh1 silencing on inhibiting OPCs proliferation following mechanical stretching was explored. As expected, the cellular viability in the Stretch group and the Ad-Control-Stretch group was significantly lower than those of the normal control untreated group (all at P<0.05). The viability of OPCs in the Ad-Cdh1-Stretch group was significantly increased as compared the Ad-Control-Stretch group but did not recover to normal control group levels (P<0. 05). This suggested that silencing Cdh1 expression significantly interfered with decreased cell proliferation activity shown to be induced by mechanical stretching (Figure 3(c)). In addition, cell cycle analysis by flow cytometry showed that interference with Cdh1 RNA expression increased the frequency of OPCs in the S phase of the cell cycle after mechanical stretching as compared with that seen in the Ad-Control-Stretch group (P<0. 05; Figures 3(d) and 3(e)). These findings suggest that Cdh1 silencing could, at least in part, promote OPCs proliferation and activation after mechanical stretch injury.

### 3.4. Possible Role of Skp2 and Id2 Expression in Cdh1-Mediated OPCs Proliferation

**Figure 4:**
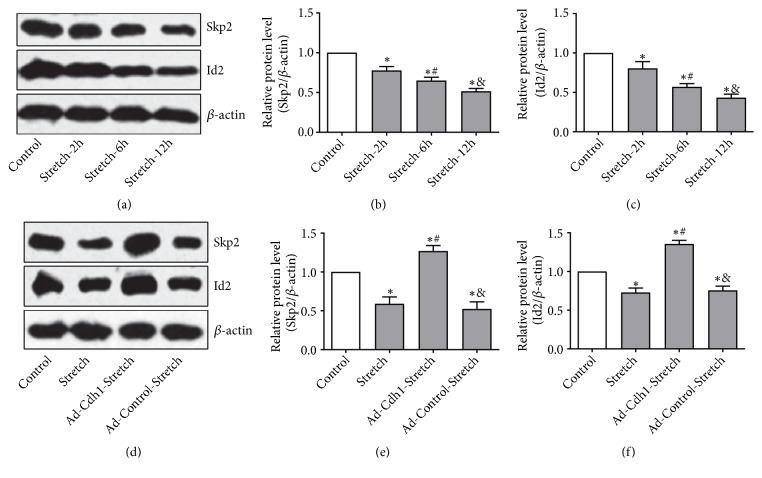
Changes in the expression of the APC-Cdh1 downstream substrates Skp2 and Id2 in OPCs after mechanical injury. (a-c) Effects of mechanical stretching on protein expression of Skp2 and Id2 in OPCs. After stretching the cells by group treatment, OPCs were cultured for 48 hours, and the total protein was assayed by Western blotting. The protein expression levels of Skp2 and Id2 after mechanical stretching were lower than those of the control group and decreased time-dependently. N = 3; *∗*P < 0.05 versus Control; #P < 0.05 versus Stretch-2-hour; and P < 0.05 versus Stretch-6-hour. (d-f) The effects of dampened Cdh1-dependent protein expression of Skp2 and Id2 in OPCs after mechanical injury. Following transduction of OPCs for 72 hours with Cdh1-shRNA adenovirus and the empty adenoviral vector, mechanical stretch injury was performed for 12 hours. Following completion of the loading, static culture was continued for an additional 48 hours. Western blotting demonstrated that protein expression of Skp2 and Id2 in the Ad-Cdh1-Stretch group was significantly increased as compared to that in the Stretch group and the Ad-Control-Stretch group. N = 3; *∗*P < 0.05 versus Control; #P < 0.05 versus Stretch; and P < 0.05 versus Ad-Cdh1-Stretch.

Expression levels of Skp2 and Id2 in the Stretch-2-hour, 6-hour, and 12-hour groups were lower than those found in the control group, and decreased gradually with extending the mechanical stretch time (all at P<0.05; Figures 4(a)–4(c)). Following Cdh1 knockdown, the expression of Cdh1 in the Ad-Cdh1-Stretch group was significantly decreased, while the protein expression levels of Skp2 and Id2 in the Ad-Cdh1-Stretch group were significantly higher than that found in the Ad-Control-Stretch group (all P<0.05; Figures 4(d)–4(f)). Observations suggest that Skp2 and Id2 might play a role in Cdh1-mediated OPCs proliferation and activation.

## 4. Discussion

We established an* in vitro* mechanical stretch injury model to simulate the pathophysiological changes of OPCs after SCI. We found that mechanical stretch injury decreased OPCs proliferation and enhanced the expression of intracellular Cdh1. Disruption of Cdh1 expression in primary OPCs promoted their postinjury proliferation and activation. Moreover, while the expression of the downstream substrates of Skp2 and Id2 were decreased, the actual expression of both Skp2 and Id2 were increased following disrupted Cdh1 expression. From these observations, APC-Cdh1 might be an important regulator of OPCs activation and proliferation after mechanical injury, a mechanism that might be dependent on targeted decreased expression of Skp2 and Id2 by ubiquitination.

In a prior report, Siebert et al. used an immunological double-labeling technique to determine an almost complete absence of BrdU/Olig1 or Ki67/Olig1 positive cells in the injured area following SCI in the rat model [12]. The results showed that OPCs proliferation was most likely derived from the injured marginal area or the normal white matter, but almost certainly not from* in situ* activated OPCs proliferation of the injured area; additionally, both necrosis and apoptosis remained as major manifestations in the center of the lesion [13]. Thus, the proliferative behavior of OPCs in the injured area was significantly inhibited as compared with the injured marginal area and at the site of normal tissue farther away from the injured site, an observation that was consistent with the conclusions of this current study. Moreover, OPCs proliferation and activation after SCI were affected by the complex internal environment of the body. This process was mainly initiated by CXCL1, IGF-1, FGF-2, and other derived signals and growth factors secreted by astrocytes and microglia [14]. We used the FX-4000T™ loading device to stretch OPCs in a model system designed to simulate* in situ* lesions after SCI, and the cells* in vitro* did not receive usual physiological signals to trigger proliferation by the functional contributions of astrocytes and microglia. Therefore, in the* in vitro* model, mechanical stretch damage to OPCs might affect its proliferative potential.

APC-Cdh1 is an important factor suppressing cell cycle transition from the G1 stage to the S stage. Cdh1 can activate APC through dephosphorylation at the late mitosis and G1 stage in proliferating cells. In addition, it can degrade the downstream cell cycle-related proteins by means of irreversible ubiquitination, so that the cells are maintained at the G1 stage, thus preventing excessive cell proliferation [15]. In this study, with the extension of stretch time, the Cdh1 expression in oligodendrocyte precursor cells was gradually increased. At the same time, flow cytometry analysis suggested that the proportion of G1 stage cells was gradually increased, while the proportion of S stage cells was gradually reduced. The mechanism might be that the increased APC-Cdh1 suppressed the signaling factors that promoted the transition of the cell cycle from the G1 stage to the S stage.

Inhibitor of DNA binding 2 (Id2) is a unique class of molecule in the helix-loop-helix (HLH) protein family and is extensively expressed in the brain of adult rats, especially in the oligodendrocyte lineage. It can phosphorylate the Rb protein or suppress the activities of cyclin-dependent kinase inhibitors (such as P21) to promote cell cycle transition from the G1 stage to the S stage [16]. Wang et al. discovered through an* in vitro* study that silencing Id2 expression with the plasmid vector could evidently inhibit the proliferation of OPCs and accelerate their differentiation [17]. Therefore, we speculated that APC-Cdh1 might block cell cycle progression from the G1 stage to the S stage in OPCs through the targeted degradation of Id2, thus restraining the proliferation and activation of OPCs after mechanical injury.

Activation of S-phase kinase associated protein 2 (Skp2) promotes cell entry into the S phase of the cell cycle and does so by decreasing the activity of the cell cycle-dependent kinase inhibitor p27 (or kip1), and its excessive activation might be associated with the reactive proliferation of astrocytes after brain injury [18]. It is formally possible that Skp2 might be recognized as a substrate by APC-Cdh1 and thus participate in ubiquitin-mediated degradation, due in part to the existence of the destruction- (D-) box motif in Skp2. Hu et al. showed that APC-Cdh1 could bind to Skp2 in the nucleus and did so via nuclear transport under the induction of the regulatory cytokine TGF-*β*, leading to decreased Skp2 expression by ubiquitination, and subsequent inhibition of cellular proliferation [19]. In this study, OPCs expression of Cdh1 was increased after mechanical stretch injury, following which, Skp2 expression decreased significantly. By contrast, adenovirus-mediated Cdh1 silencing enhanced Skp2 expression by OPCs following injury. This observation indicated that Skp2 might indeed be the direct downstream substrate of APC-Cdh1 in the pathological process of mechanical spinal cord injury. We speculated that enhanced Cdh1 expression in OPCs after injury might block the cell cycle in a mechanism that might be in part dependent on ubiquitin-mediated degradation of Skp2, a process that might block the activation and proliferation of OPCs after injury.

In our prior* in vivo* study, we found that the mRNA expression of Cdh1 in the cortical somatosensory motor region was significantly increased after SCI in rats. Decreased Cdh1 expression by RNA interference stimulated the regeneration of damaged axons[20]. These results suggested that APC-Cdh1 was involved in axonal repair following SCI, and its mechanism might be related to the degradation of downstream substrates SnoN and Id2 [21, 22]. In combination with the comprehensive analysis reported in this study, APC-Cdh1 was not only an important pathway in regulating axonal regeneration after SCI, but might also serve as an intracellular factor that disrupted activation and proliferation of OPCs. APC-Cdh1 was the cross-point between axonal regeneration and the activation and proliferation of OPCs. Thus, exploration of the effects of regulating APC-Cdh1 activity on OPCs activation and proliferation could prove to be quite significant in terms of understanding the mechanisms of protecting the spinal cord after injury.

Observations made in the current study add to the growing body of literature that have revealed novel functions of APC in the CNS including cell cycle regulation, axonal guidance, synaptic plasticity, neurogenesis, and survival of neuronal cells. Interestingly, others have found that degradation products of APC-targeted substrates are associated with neurodegenerative conditions, including Alzheimer's disease, an observation that might implicate dysregulation of APC in neurodegenerative diseases [23], and highlighting the role of APC-Cdh1 as a potential therapeutic target in neuronal degeneration and injury. Therapeutic targeting of Cdh1 also has a historically important context, since it was shown more than a decade ago that inhibition of Cdh1, a coactivator of the APC/cyclosome, in embryonic primary cultures promotes axon elaboration and override growth suppressing activities that are mediated by soluble myelin-derived factors [24]. Furthermore, recognizing that demyelination is a key initiating event in SCI and that oligodendrocyte apoptosis plays an important role in triggering neuronal demyelination, Huang et al. developed a compressed SCI rat model [25]. This group set out to determine whether or not demyelination and oligodendrocyte apoptosis are seen following compressed SCI. Neuronal demyelination occurred shortly after compressed SCI and was provoked, at least in part, by oligodendrocyte apoptosis, a process that was associated with enhanced expression of Id2 after compressed SCI in the rat model [25].

## 5. Conclusions

In conclusion, mechanical stretch injury was shown to dampen the activation and proliferation of OPCs in a process that was accompanied by high intracellular expression of Cdh1. The downregulation of Cdh1 expression by RNA interference stimulated activation and proliferation of OPCs after mechanical injury. The present study indicates that APC-Cdh1 might serve as a potential therapeutic target in the settings of both recovery and regeneration of neural function after SCI.

## Figures and Tables

**Table 1:** Sequences of primers used for real-time PCR.

Primer	5′-3′ Sequence (Forward; Reverse)
Cdh1	For: ACTCCGCACTGCTGAAGAAT
	Rev: GCTTGCTGCTGAGGGAATAC
Skp2	For: TTGGAGGTGGTCTTTTCTGG
	Rev: TCTCCTGCTCTGGTTGTGTG
Id2	For: TTTCCTCCTACGAGCAGCAT
	Rev: CCAGTTCCTTGAGCTTGGAG
*β*-actin	For: GTCAGGTCATCACTATCGGCAAT
	Rev: AGAGGTCTTTACGGATGTCAACGT

## Data Availability

The data set supporting the results of this article are included within the article.
